# Huatuo Zaizao pill ameliorates cognitive impairment of APP/PS1 transgenic mice by improving synaptic plasticity and reducing Aβ deposition

**DOI:** 10.1186/s12906-018-2237-2

**Published:** 2018-05-29

**Authors:** Jing-Hua Zhang, Lin-Jie Yu, Hui Yang, Zhen Hui, Su Jiang, Ling Chen, Yang Zhao, Su-Lei Wang, Yi Liu, Yun Xu

**Affiliations:** 1 Department of Neurology, Nanjing Drum Tower Hospital Clinical College of Traditional Chinese and Western Medicine, Nanjing University of Chinese Medicine, Nanjing, 210008 Jiangsu People’s Republic of China; 2 Department of Neurology, Drum Tower Hospital, Medical School of Nanjing University, 321 Zhong Shan Road, Nanjing, 210008 Jiangsu People’s Republic of China; 3 Department of Neurology, The Third Affiliated Hospital of Nanjing University of Chinese Medicine, Nanjing, 210001 Jiangsu People’s Republic of China; 4 Department of Physiology, Nanjing Medical University, Nanjing, 211166 Jiangsu People’s Republic of China; 5 The State Key Laboratory of Pharmaceutical Biotechnology, Nanjing University, Nanjing, 210008 Jiangsu People’s Republic of China; 6 Jiangsu Key Laboratory for Molecular Medicine, Nanjing University Medical School, Nanjing, 210008 Jiangsu People’s Republic of China; 7 Nanjing Neuropsychiatry Clinic Medical Center, Nanjing, 210008 People’s Republic of China

**Keywords:** Huatuo Zaizao pill, Alzheimer’s disease, Amyloid-β, Synaptic plasticity

## Abstract

**Background:**

It is well known that Alzheimer’s disease (AD) is a progressive neurodegenerative disease characterized by memory deficits and cognitive decline. Amyloid-β (Aβ) deposition and synaptic dysfunction play important roles in the pathophysiology of Alzheimer’s disease (AD). The Huatuo Zaizao pill (HT) is a Traditional Chinese Medicine (TCM) that has been used clinically for many years in China, mainly for post-stroke rehabilitation and cognitive decline; however, the mechanism of cognitive function is not clear. In this study, we investigated the effect of HT on hippocampal synaptic function, Amyloid-β (Aβ) deposition in APP/PS1 AD transgenic mice.

**Method:**

Six-month-old APP/PS1 transgenic (Tg) mice were randomly divided into control, HT-treated, and memantine (MEM)-treated groups. Then, these groups were orally administered vehicle (for the control), HT (0.25 g/kg) and MEM (5 mg/kg) respectively for 4 weeks. The Morris water maze, Novel Object Recognition, and Open field tests were used to assess cognitive behavioral changes. We evaluated the effects of HT on neuronal excitability, membrane ion channel activity, and synaptic plasticity in acute hippocampal slices by combining electrophysiological extracellular tests. Synaptic morphology in the hippocampus was investigated by electron microscopy. Western blotting was used to assess synaptic-associated protein and Aβ production and degrading levels. Immunofluorescence staining was used to determine the relative integrated density.

**Results:**

HT can ameliorate hippocampus-dependent memory deficits and improve synaptic dysfunction by reversing LTP impairment in APP/PS1 transgenic mice. Moreover, HT reduces amyloid plaque deposition by regulating α-secretase and γ-secretase levels.

**Conclusion:**

HT can improve the learning and memory function of APP/PS1 transgenic mice by improving synaptic function and reducing amyloid plaque deposition.

**Electronic supplementary material:**

The online version of this article (10.1186/s12906-018-2237-2) contains supplementary material, which is available to authorized users.

## Background

Alzheimer’s disease (AD) is the most common type of dementia. AD is a chronic degenerative disease of the primary central nervous system, characterized by progressive cognitive dysfunction and memory impairment. The incidence of AD has increased annually, and it is currently estimated that more than 10% of the elderly are suffering from AD. As such, AD has become the third leading cause of death in the elderly [1]. Currently, drugs for the treatment of AD consist of cholinesterase inhibitors and N-methyl-D-aspartate receptors (NMDA) antagonists; however, these drugs cannot control or reverse the progression of AD. Moreover, some medicines have certain side effects. Therefore, we need to explore new drugs to slow down cognitive decline.

The deposition of Aβ leads to the destruction of synaptic signaling pathways and the destruction of dendritic spines, which affects synaptic morphology and function, leading to memory and behavioral defects [2]. Many studies suggest that the protection of synapses can diminish the observed memory deficits in AD [3]. Long-term potentiation (LTP) is widely considered to be one of the major cellular mechanisms of learning and memory [4], which has received much attention in Alzheimer’s disease (AD), a neurodegenerative disease that causes obvious cognitive decline and dementia. The accumulation of Aβ is considered to be a main pathogenic feature of AD. Studies have found that Aβ levels increase in the brain of AD patients and AD mouse models. Aβ accumulation may cause multiple neuronal damage and lead to cognitive impairment [5].

The Huatuo Zaizao pill (HT), a pure natural preparation derived from plants, which is a TCM formulation marketed in China [6]. HT was listed in the pharmacopoeia of the People’s Republic of China Part I (Pharmacopoeia Commission of the People’s Republic of China, 2015), as HT can be used to promote rehabilitation after stroke. HT was widely used in the treatment of cerebrovascular diseases, stroke and hemiplegia [7–9]. Meta-analysis showed that HT promoted the functional recovery in patients with ischemic stroke [10, 11]. HT treatment could promote functional recovery by enhancing the expression of brain-derived neurotrophic factor (BDNF) and increasing the level of neurogenesis in cerebral I/R animal [12]. HT mainly composed of five Chinese herbs: *Ligusticum chuanxiong hort (Umbelliferae), Evodia rutaecarpa (Juss.) Benth, Carthamus tinctorius L. (Safflower), and Angelica sinensis (Oliv.) Diels, Borneol.* Researches have shown that HT have many pharmacological effects, which could improve the cognitive decline [13]. HT combined with donepezil improved the cognitive function in the treatment of subcortical ischemic vascular dementia [14]. Another study showed that HT improved the degree of cognitive function and memory impairment by improving blood hypercoagulability and microcirculation [15]. HT contains many ingredients, such as Ferulic acid (FA), Evodin, Evodiamine and Rutaecarpine, especially FA has a therapeutic effect in the treatment of AD. FA reduced the progression of cognitive deficits in animal models [16–18].

Although some clinical reports indicated that HT can be used to treat cognitive decline, but there is no study on its mechanism of action. In our study, we hypothesize that HT alleviates cognitive impairments. As one of the mouse models of AD, APPswe/PS1dE9 (APP/PS1) co-expressing the delta exon 9 variant of presenilin 1 (PS1) and the Swedish mutation of β-amyloid precursor (APP) [19, 20]. Additionally, APP/PS1 transgenic mice have developed a few Aβ plaques and began to have deficits in learning and memory at 6 months of age compared to wild-type (WT) mice [21–23]. Thus, in this study, we showed whether HT could attenuate memory impairment in the APP/PS1 mice and investigate the molecular mechanisms involved in the protection of synapses.

## Methods

### Drugs

HT was obtained from GuangZhou BaiYun Shan QiXing Pharmaceutical Co, Ltd. (Guangzhou, China; batch number: 17121), and placed in 4 °C. The dosage for clinical patients is 8–16 g per day. HT quality management data can be found in the pharmacopoeia of the People’s Republic of China Part I (Pharmacopoeia Commission of the People’s Republic of China, 2015). The HT solution was dissolved in 0.9% physiological saline with HT powder (0.0625 g/ml).

### Animals

Six-month-old male APP/PS1 transgenic mice which have memory deficits (provided in the Additional file 1: Figure S1) were provided by the Model Animal Research Center of Nanjing University in China. The mean body weight was 36 ± 5.2 g. All animals were housed in standard cages under 12 h/12 h light/dark conditions with free access to food and water. All animal experimental procedures were approved by the Animal Care Committee of Nanjing University and conformed to the Animal Ethical Standards.

### Grouping and treatment

Animals were placed into each group using a randomized block design. The selected HT dosages (0.25 g/kg) were based on our previous experiment (provided in the Additional file 2: Figure S2). Mice were divided into the following groups: APP/PS1 group, HT at 0.25 g/kg group, and MEM at 5 mg/kg group. HT and MEM were dissolved in 0.9% saline. The APP/PS1 group was given the same volume of saline. The treatments were administered intragastricly once daily for 4 weeks. At the end of the fourth week, The Morris water maze, Novel Object Recognition, and Open-field tests were used to assess cognitive behavioral changes. The mice were anesthetized depth with sodium pentobarbital (50 mg/kg) and killed by decapitation in an unconscious state [24]. Then the brains were harvested for electrophysiology, Golgi Staining, histopathological, Immunofluorescence staining, and Western blotting assessments.

### Morris water maze

The cognitive function of the animals was evaluated using a Morris water maze as previously described [25]. The Morris water maze (MWM) test was administered before treatment (to confirm behavioral deficits in APP/PS1 mice) and after the HT, MEM and saline treatments. In brief, mice can escape onto the platform within 60 s and then stay on the platform for at least 5 s during acquisition phase trials (days 1–5). If the mice failed to find the platform within the permitted 60 s, they were manually placed on the platform for 30 s so that they could recollect the location of the platform, and the latency was recorded for 60 s. The escape distance and escape latency were analyzed by Any-maze software (Stoelting, USA). On the 6th day, the platform was removed from the water maze, and the mice were released to swim freely for 60 s. The number of times they crossed the target platform and the time spent in the target quadrant were automatically recorded by the video/computer system.

### Novel object recognition

The Novel Object Recognition test is conducted in an open field arena. During habituation, the mice are allowed to explore an empty arena. Three days after habituation, the mice were presented with two similar objects during the first session. Then, one of the two objects was replaced by a new object during a second session. The time spent exploring each object and the discrimination index percentage were recorded. This test can be used to assess the cognitive impairment of transgenic mice and to assess the impact of new objects on their cognition.

### Open field test

The open field test (OFT) is primarily used to observe a variety of behaviors after the mouse is released into the open environment and reflects the neuropsychiatric activities of the experimental animal. Spontaneous open field experiments can observe the spontaneous activities of mice. The experimental device was a square Plexiglas box, and the wall was a brown color. A video tracking system was fixed above the experimental platform. The mice were placed in the same position in the box, and the total distance the mice traveled within 5 min was recorded. The field instrument was cleaned up after the experiment to avoid leaving an abnormal odor. The following observations were made: the total distance, central time, corner time, and the trajectories of the mice.

### Electrophysiology

Hippocampal CA1 long-term potentiation (LTP) was recorded as described previously [26]. Transverse slices of the hippocampus (400 mm) were cut from the mouse brain. The brains were rapidly removed after decapitation and maintained in cold oxygenated (95% O2/5% CO2) physiological media. The hippocampal slices were continuously perfused with artificial cerebrospinal fluid (ACSF) composed of the following: NaCl 124 mM, CaCl2 2.0 mM, KCl 4.5 mM, MgCl2 1.0 mM, NaHCO3 26 mM, NaH2PO4 1.2 mM, D-glucose 10 mM, and pH 7.4. The orthorhombic stimulus was used to stimulate the Schaffer collateral/connective pathway using a polished bipolar tungsten electrode. The stimulator (SEN-3301, Nihon Kohden, Japan) provides a constant current pulse (0.1 ms, 0.05 Hz). The excitatory postsynaptic potential (EPSP) was recorded from the radiation layer of the CA1 region and connected to a preamplifier with a high pass pulse of 5 kHz. The signal was amplified using a different AC amplifier (A-M Systems, 1700, Seattle, WA). EPSP was digitized using the pCLAMP system (Axon Instrument Inc, Foster City, CA). The input/output (I/O) function was measured by the average EPSP slope and the stimulus intensity of 0.1–1.1 mA. Long-term enhancement (LTP) is generated by high frequency stimulation (HFS, 100 Hz, pulse 100 Hz) with the same intensity as the previous HFS. Subsequently, the single pulse recording was resumed and continued for 60 min. When the EPSP slope is more than 60 min longer than 20% of the baseline, it is considered to be LTP induced.

### Golgi staining for dendritic spines

According to the manufacturer’s instructions, Golgi staining was performed using a fast Golgi staining kit (FD Neurotechnologies, Elliot City, MD, USA). The brain tissues were mixed with solution A and B at room temperature for 2 weeks in the dark and then transferred to solution C at 4 °C for at least 2 days and up to 7 days. Subsequently, the samples were placed in a 200-μm slice by a cryostat and placed on a microscope slide. The sections were rinsed twice for 4 min each with a double distilled water or Milli-Q water. The slices were placed in a mixture containing 1 part of solution D, 1 part of solution E and 2 parts double distilled water or Milli-Q water. Then, Golgi Staining was performed as described previously [6]. The slides were dehydrated and cleared, and a coverslip was placed.

### Western blotting

Proteins were isolated from the hippocampus of brain according to the protein extraction kit instructions (Beyotime Biotechnology, China). Protein concentrations were determined using the BCA protein assay kit (Bioworld, USA). Equal volumes of protein were separated using 10% dodecyl sulfate, sodium salt polyacrylamide gel electrophoresis (SDS-PAGE) and transferred to polyvinylidene fluoride (PVDF) membranes. Membranes were blocked in 5% non-fat milk for 1 h and incubated overnight at 4 °C with primary antibodies as follows: 6E10 (1:1000; Covance, USA), anti-APP (1:1000, Abcam Technology, UK), anti-PSD95 (1:1000, Cell Signaling Technology, USA), anti-SYN (1:1000, Abcam Technology, UK), anti-IDE (1:1000,Abcam Technology, UK), anti-ADAM10 (1:1000, Millipore Technology, USA), anti-BACE1 (1:1000, Millipore Technology, USA), anti-PS1 (1:1000, Cell Signaling Technology, USA), and anti-GRPDH (1:2000, Cell Signaling Technology, USA). Membranes were rinsed with Tris-buffered saline with Tween 20 (TBST) three times. Then, membranes were incubated with HRP-conjugated anti-rabbit or anti-mouse secondary antibodies for 2 h. Protein signals were visualized with the chemiluminescence reagents provided with the ECL kit (Bioworld, USA), and quantitation of proteins was determined by densitometric analysis using ImageJ software (Bio-Rad, Hercules, USA).

### Hematoxylin-eosin staining (HE) staining

HE staining was used to observe the pathologic changes in the hippocampal CA1 and cortex areas in APP/PS1 mice. The brain slices embedded in paraffin were cut into 4-μm slices. The brain slices were dewaxed in xylene and dehydrated by fractional alcohols. Then, sections of each group were stained with HE substance cresyl violet. The neurons in the hippocampal CA1 and cortex were examined using an Olympus optical microscope.

### Immunofluorescence staining

Immunofluorescence staining was performed as described previously [27]. After anesthesia, the mice were perfused with 0.9% saline and 4% paraformaldehyde. The brain was removed and placed in 15 and 25% sucrose solution. Subsequently, the brain was cut into 40 μm slices using a low temperature thermostat (Leica, Germany). Each section was incubated with 10% normal sera, followed by overnight incubation with the primary antibodies, 6E10 (1:500, Covance, Princeton, NJ, USA), at 4 °C. Then, sections were incubated with the appropriate secondary antibodies for 2 h at room temperature in the dark. The Aβ antibody showed dense amyloid deposits in the cortex and hippocampus of APP/PS1 mice with an Olympus microscope and a BX51 digital camera (Olympus, Japan). The images were quantified with IPP 6.0 software. All measurements were performed in a randomized and blinded manner.

### Statistical analysis

The results were expressed as the mean ± SEM and analyzed using SPSS 18.0 statistical analysis software (SPSS, USA). Group differences in escape latency were analyzed using two-way analysis of variance (ANOVA) with repeated measures followed by Bonferroni multiple comparison test with day and treatment as the sources of variation. One-way ANOVA and Bonferroni’s post hoc were performed to analyze other data. *P* values < 0.05 were considered statistically significant.

## Results

### HT ameliorate learning and memory deficits in APP/PS1 transgenic mice

**Fig. 1:**
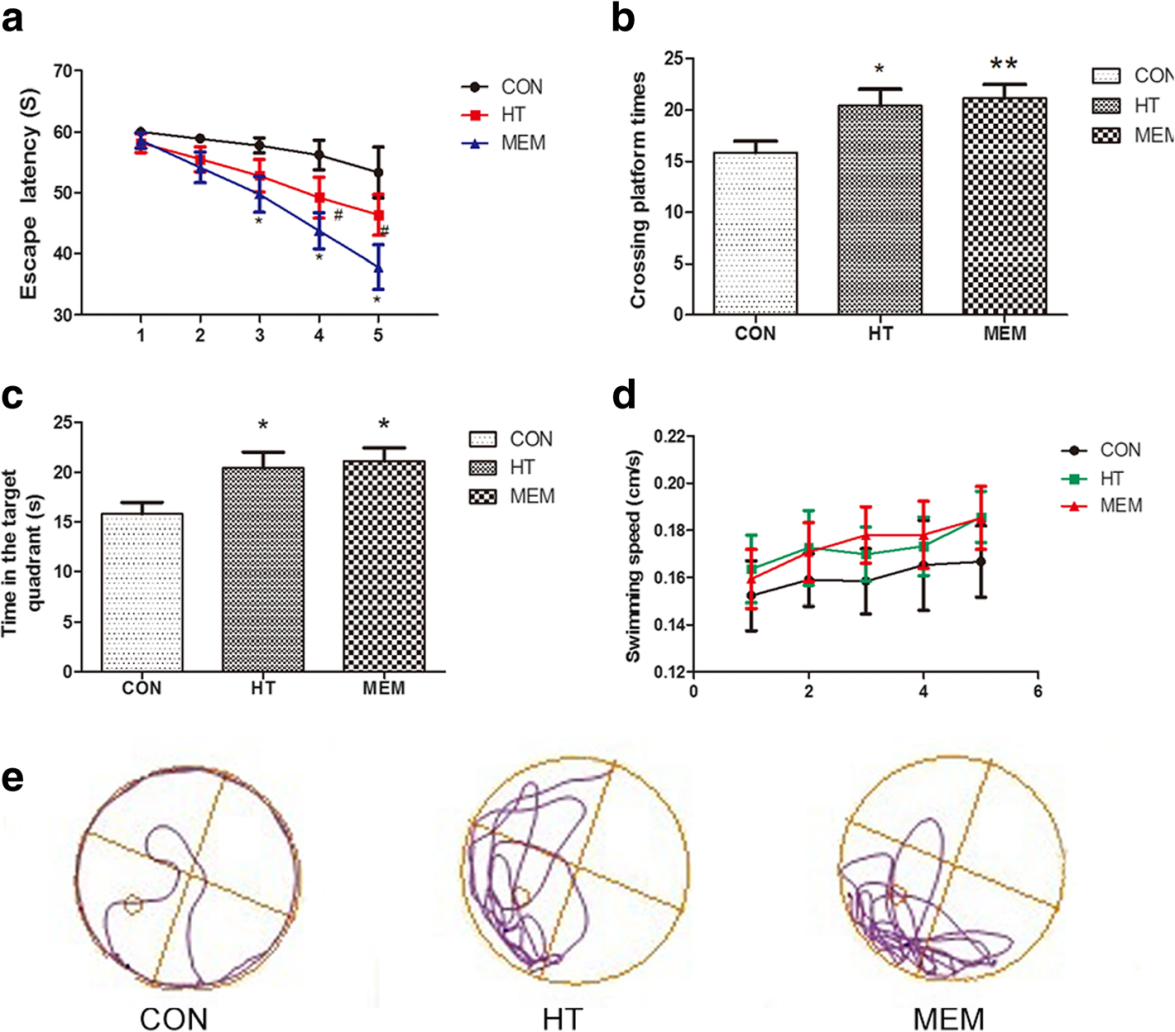
HT Ameliorates Learning and Memory Deficits in APP/PS1 Transgenic Mice. HT improves cognitive impairment in APP/PS1 Transgenic Mice. **a** Escape latency for escape to the submerged platform in the probe trails. **b** Crossing platform times in the probe trails. **c** The time in the target quadrant. **d** Swimming speed in the training trails. **e** The track of probe test. Data are presented as the mean ± SEM (*n* = 15). *P < 0.05, **P < 0.01, compared with control group; #*P* < 0.05, compared with control group. One-way ANOVA followed by post hoc Bonferroni’s test; *P < 0.05 compared with control group, #*P* < 0.05 compared with control group

**Fig. 2:**
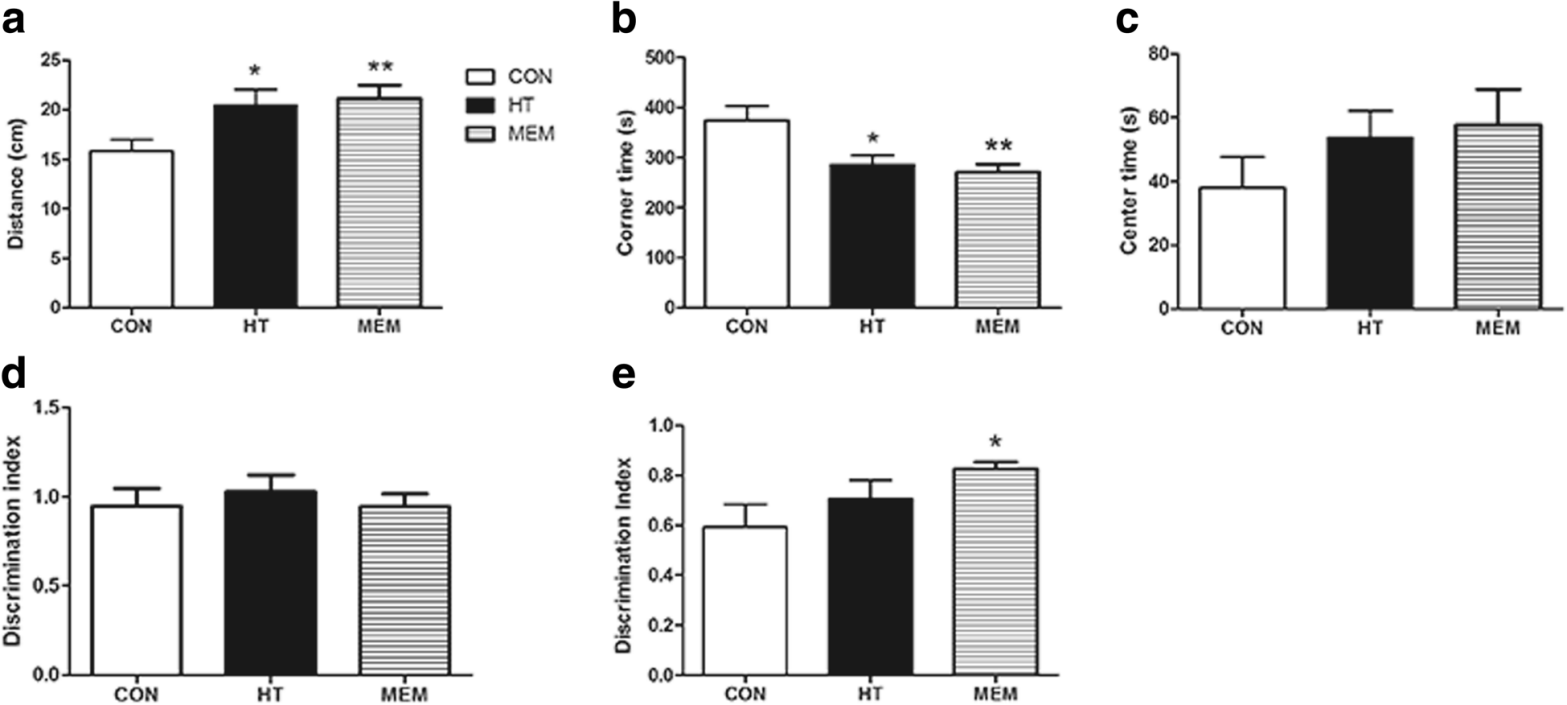
HT ameliorates cognitive impairment in APP/PS1 Transgenic Mice. **a** Total distance in the open field test. **b** Corner times in the open field test. **c** Center times in the open field test. **d** Discrimination index during acquisition memory phase (training) during the novel object recognition test, showing no significant differences between the different groups. **e** Discrimination index for novel-object recognition during the retention memory phase (test). HT and MEM could rescue memory deficits and relieve anxiety in APP/PS1 mice. Values are expressed as the mean ± SEM *n* = 15 in each group; *P < 0.05 compared to the control group; **P < 0.01 compared with control group

As shown in Fig. 1, compared to the control group (APP/PS1), HT can significantly shorten escape latency, increase crossing platform times, and increase the time in the target (*P* < 0.05). There were no significant differences in escape latency and crossing platform times between the HT group and the MEM group (*P* > 0.05) (Fig. 1a and b). Although MEM can increase the time in the target significantly (*P* < 0.01) (Fig. 1c), there were no significant difference between the HT groups and the MEM groups (*P* > 0.05). In addition, there were no differences in swimming speed among the three groups (*P* > 0.05) (Fig. 1d). Subsequently, open field and novel object tests were conducted for further study. In the open field test, there were significant differences in total distance and the times in corner, with HT and MEM groups performing better than the APP/PS1 mice (*P* < 0.05) (Fig. 2a and b). There were no significant differences in the center times among the three groups (*P* > 0.05) (Fig. 2c). In the Novel Object Recognition test, the discrimination index of MEM-treated APP/PS1 mice was higher than in the control APP/PS1 mice (*P* < 0.05; Fig. 2e). Although HT can increase the discrimination index, there is no statistical significance (*P* > 0.05; Fig. 2e). Above all, the results suggested that the learning memory, and spatial localization ability of APP/PS1 mice was improved significantly between the HT groups and the MEM groups.

### Pathological morphological changes in the hippocampus CA1 and cortex of APP/PS1 transgenic mice

**Fig. 3:**
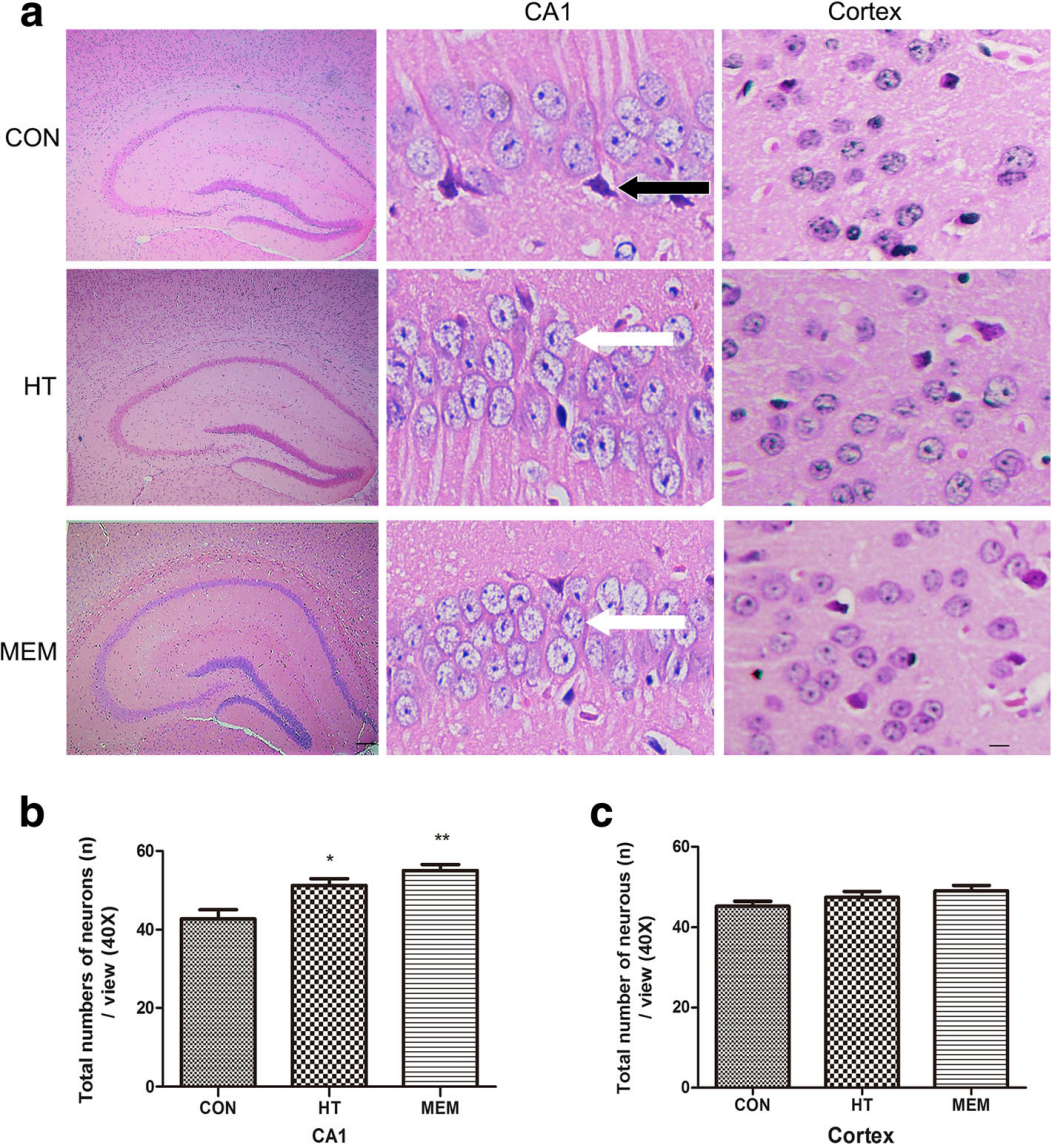
Effect of HT on the histopathological changes in the hippocampus and cortex of APP/PS1 Transgenic Mice. **a** HE-stained images in the hippocampal CA1 and cortex of APP/PS1, HT and MEM groups. Scale bar = 200 μm. Injured neurons in APP/PS1 mice are darkly stained and exhibited shrunken and triangulated neuronal body (black arrows). Cells are arranged indisorder with a slightly changed cell polarity, neuron loss can also be seen. By contrast, treatment with HT and MEM significantly inhibited the histopathological damage (white arrows). **b** The hippocampal CA1 in the HT and MEM mice contained significantly more total neurons than did that in the APP/PS1 mice. **c** The total numbers of neurons in cortex did not significantly differ among the three groups. Data are presented as the mean ± SD. *P < 0.05, **P < 0.01 versus control group. Scale bar = 20 μm, *n* = 6 per group

HE staining (Fig. 3) was conducted to assess effects of HT on neuronal loss in AD brain. In APP/PS1 mice, cells were arranged in disorder with a slightly changed cell polarity, there was a few cells that showed signs of neurodegeneration, such as darkly stained and exhibited shrunken and triangulated neuronal body. The neuron morphology in the hippocampal CA1 area of the HT and MEM groups demonstrated a certain degree of improvement, the neurons displayed the same morphological characteristics, cells arranged closely, the cell membrane, cytoplasm and nuclear membrane structure were clearer. In addition, we found that the neuronal number of CA1 in the HT and MEM group were higher than that in the APP/PS1 group (Fig. 3b, *P* < 0.05). However, there were no significant differences in the neuronal number of cortex among three groups. (Fig. 3c, *P* > 0.05).

### HT improves synaptic function in APP/PS1 transgenic mice

**Fig. 4:**
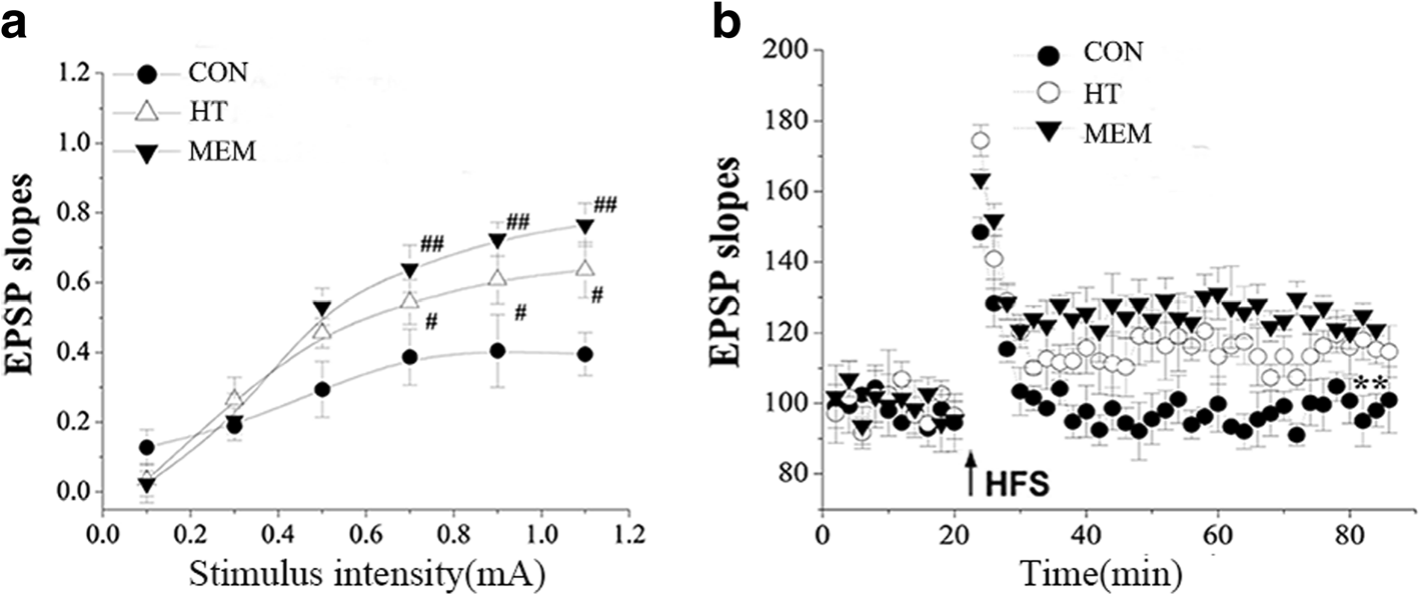
HT improves synaptic function in APP/PS1 Transgenic Mice. Electrophysiological determination of deficits in Schaffer collateral-CA1 synaptic transmission and the induction of long-term potentiation. **a** A plot showing input/output (I/O) curves in response to stimulus (0.1–1.1 mA) at the hippocampal CA1 region of APP/PS1 control mice, APP/PS1 mice receiving HT, and APP/PS1 mice receiving MEM treatment (6 months old). The I/O curve was measured by averaging the slope of EPSPs against stimulus intensity at 0.1–1.1 mA. The slope of the regression line for the I/O curve in APP/PS1 mice was markedly reduced. With HT or MEM treatment, the reduction in the I/O curve slope was significantly improved in APP/PS1 mice. All values are expressed as the mean ± SEM from 6 mice in each group. #*P* < 0.05 HT group versus APP/PS1 control mice, ##*P* < 0.01 MEM group versus APP/PS1 control mice. **b** The capacity to establish long-term potentiation (LTP) from high-frequency stimulus (HFS, 100 pulses at 100 Hz) with the same pre-HFS intensity at hippocampal CA1 region of APP/PS1 control mice. APP/PS1 mice with HT treatment and APP/PS1 mice with MEM treatment

Long-term potentiation (LTP) induction was one of the main manifestations of synaptic function. Whether HT treatment enabled neurons to establish more elective LTP was examined. Using brain slices from APP/PS1 mice and with HT and MEM treatment, we found that the slope of the I/O curve obtained from APP/PS1 mice (B = 0.38; *n* = 12 slices/6 mice) was significantly less than in HT APP/PS1 mice and MEM APP/PS1 mice (B = 0.51; *P* < 0.05, n = 12 slices/6 mice; B = 0.62, *P* < 0.01, *n* = 12 slices/6 mice) (Fig. 4a). Stable LTP (102.43 ± 5.18%; *n* = 12 slices/6 mice, Fig. 4b) was induced in APP/PS1 mice, and the mice failed to be stably stimulated by high frequency (HFS, 100 pulses at 100 Hz). The results showed that HT and MEM could improve synaptic dysfunction by protecting the APP/PS1 mice from LTP impairment (116.45 ± 4.55%; *n* = 12 slices/6 mice; 126.35 ± 3.45%; *n* = 12 slices/6 mice, Fig. 4b).

**Fig. 5:**
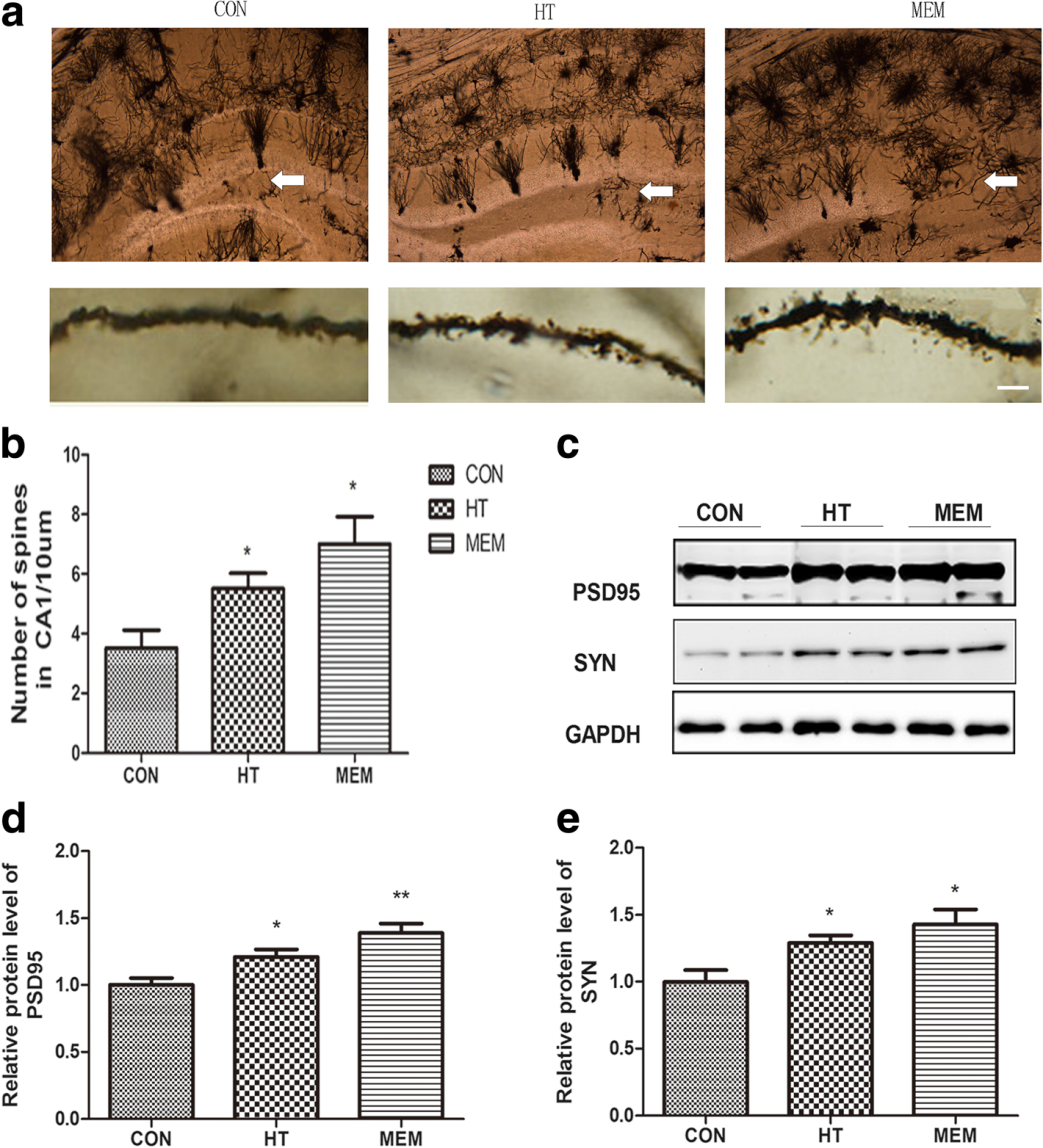
HT improves synaptic function in APP/PS1 Transgenic Mice. **a** Golgi staining of the hippocampal area(top), HT mediated preservation of the number of spines per given dendritic length in the hippocampus CA1. Scale bar = 10 μm. **b** Quantitative analysis of spine density in CA1. **c** The PSD95 and SYN protein levels were measured by Western blotting in the different groups. **d** Quantitative analysis of PSD95 expression. *P < 0.05, **P < 0.01 versus control group. **e** Quantitative analysis of SYN expression. *P < 0.05 versus control group, *n* = 6 per group

The development of dendrites and the number of dendritic spines can both affect synaptic function in AD mice [28]. Here, we used Golgi staining to examine the effects of HT on dendritic morphology. As shown in Fig. 5a, to further characterize the differences in dendritic morphology between groups, we examined the density of dendritic spines. The hippocampus of APP/PS1 mice exhibited a lower density of spines, and this reduction could be rescued by HT and MEM (*P* < 0.05) (Fig. 5b).

To explore the mechanism of HT on protecting synapse APP/PS1 transgenic mice, we detected the expression of synaptic related proteins in the hippocampus of APP/PS1 mice, including Postsynaptic density protein 95 (PSD95) and Synaptophysin (SYN). The expressions of PSD95 and SYN were significantly increased in APP/PS1 mice treated with HT and MEM compared to the APP/PS1 mice treated with saline (*P* < 0.05) (Fig. 5c-e).

### HT decreased Aβ plaque deposition in APP/PS1 mice

**Fig. 6:**
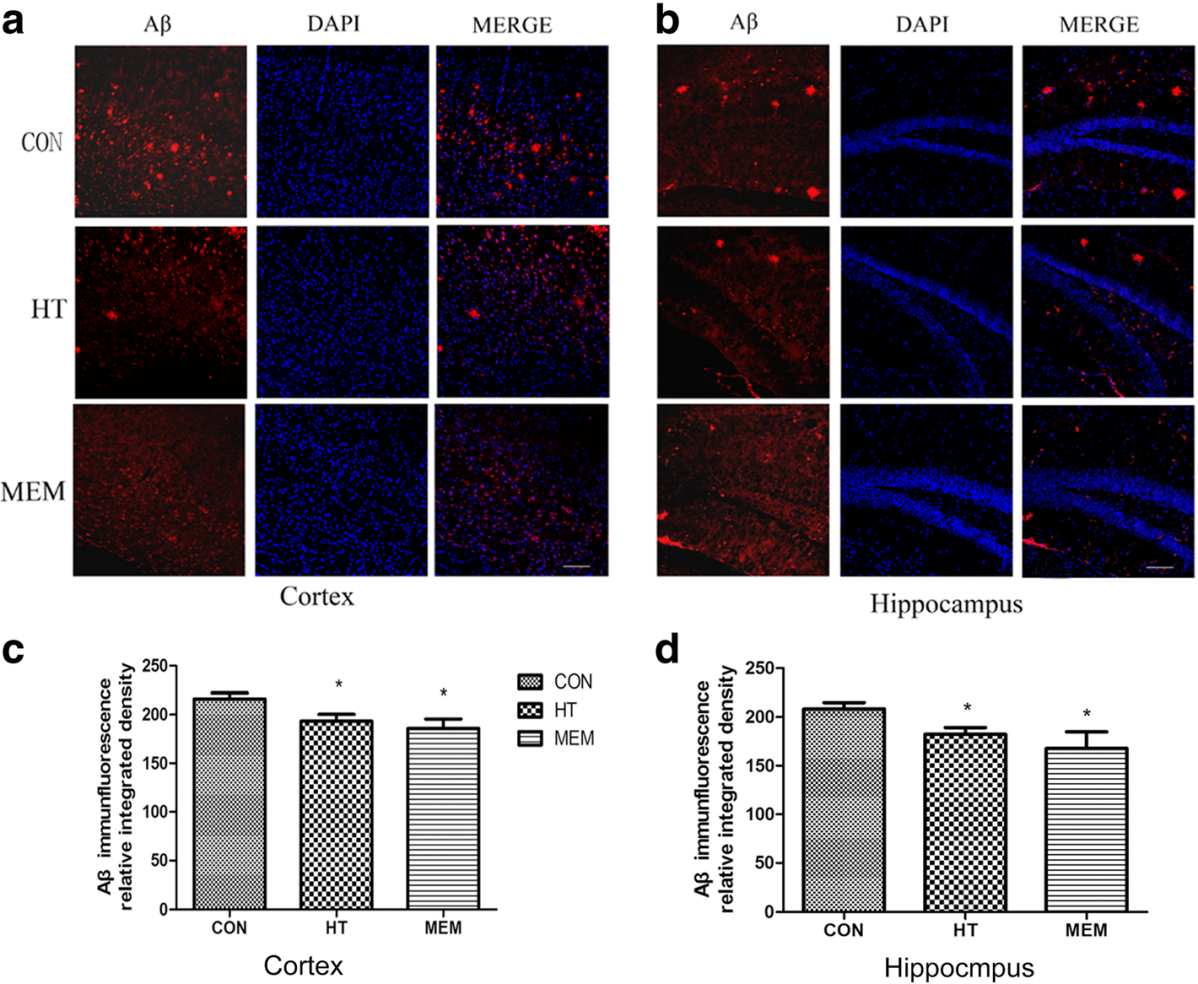
HT decreased Aβ plaque deposition in the brains of APP/PS1 mice. Immunofluorescence for Aβ in the cortex (**a**) and hippocampus (**b**) of the APP/PS1 mice, with HT treatment and MEM treatment of APP/PS1 mice. Scale bar = 100 μm. The relative integrated density of Aβ in the cortex and hippocampus between different groups, HT-treated and MEM-treated groups better than the control group. (**c** and **d**), *n* = 6 per group

**Fig. 7:**
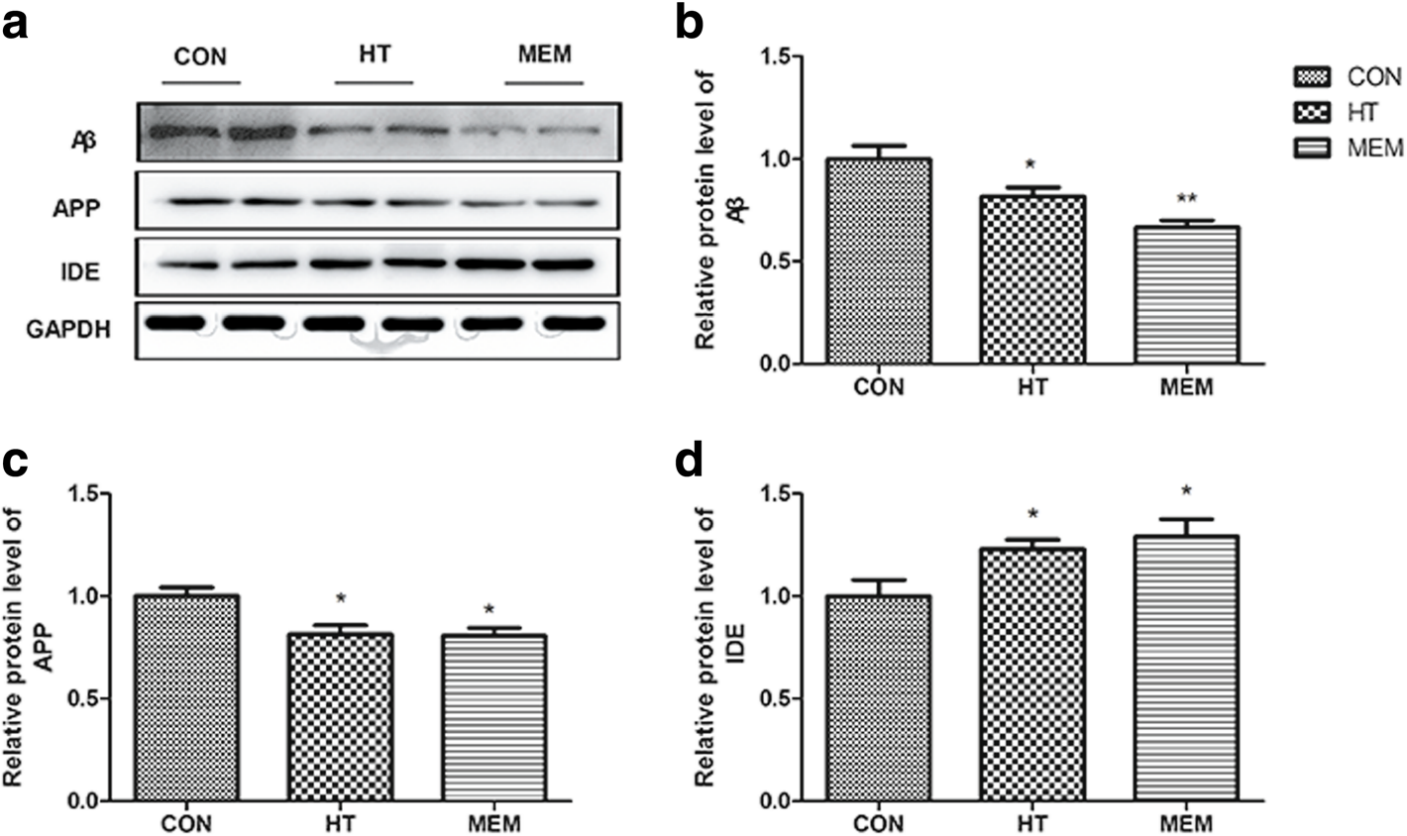
HT reduced APP expression and upregulated IDE expression in the brains of APP/PS1 mice. **a** The protein levels of Aβ, APP and IDE were measured by Western blotting in the different groups. **b** Quantitative analysis of Aβ expression. *P < 0.05 versus control group. **c** Quantitative analysis of APP expression. *P < 0.05 versus control group. **d** Quantitative analysis of IDE expression. *P < 0.05 versus control group, *n* = 6 per group

Aβ accumulation and plaque deposition may induce synaptic failure, dendritic and axonal atrophy, and neuronal death [29, 30]. HT seemed to promote the structural synaptic integrity, so we investigated if this effect could ameliorate Aβ deposition in APP/PS1 mice. Our study demonstrated that HT and MEM could lower Aβ deposition both in the hippocampus and cerebral cortex compared to the control-treated group in the APP/PS1 mice (*P* < 0.05) (Fig. 6a-d). However, there are no differences between the HT group and MEM group (*P* > 0.05) (Fig. 6c and d). The Aβ levels in the hippocampus of APP/PS1 mice were further confirmed with a Western blot. Similar to the immunofluorescence staining, the levels of Aβ were significantly reduced in the hippocampus (Fig. 7a and b) of 6-month-old APP/PS1 mice treated with HT and MEM compared to saline-treated APP/PS1 mice.

### HT reduces APP expression and upregulates insulin-degrading enzyme (IDE) expression in the brains of APP/PS1 mice

APP is the precursor protein that leads to Aβ plaque formation associated with AD. We measured APP expression with Western blotting methods (Fig. 7a). HT and MEM treatment significantly decreased the APP protein level in the hippocampus of the APP/PS1 mice compared to the saline-treated group (*P* < 0.05). There are no differences in the APP expression between the HT and MEM groups (*P* > 0.05) (Fig. 7c). In addition, the HT and MEM groups could upregulate IDE expression better than the saline-treated APP/PSI mice (*P* < 0.05) (Fig. 7d).

### Effects of HT on α-secretase, β-secretase, and γ-secretase enzyme activities

**Fig. 8:**
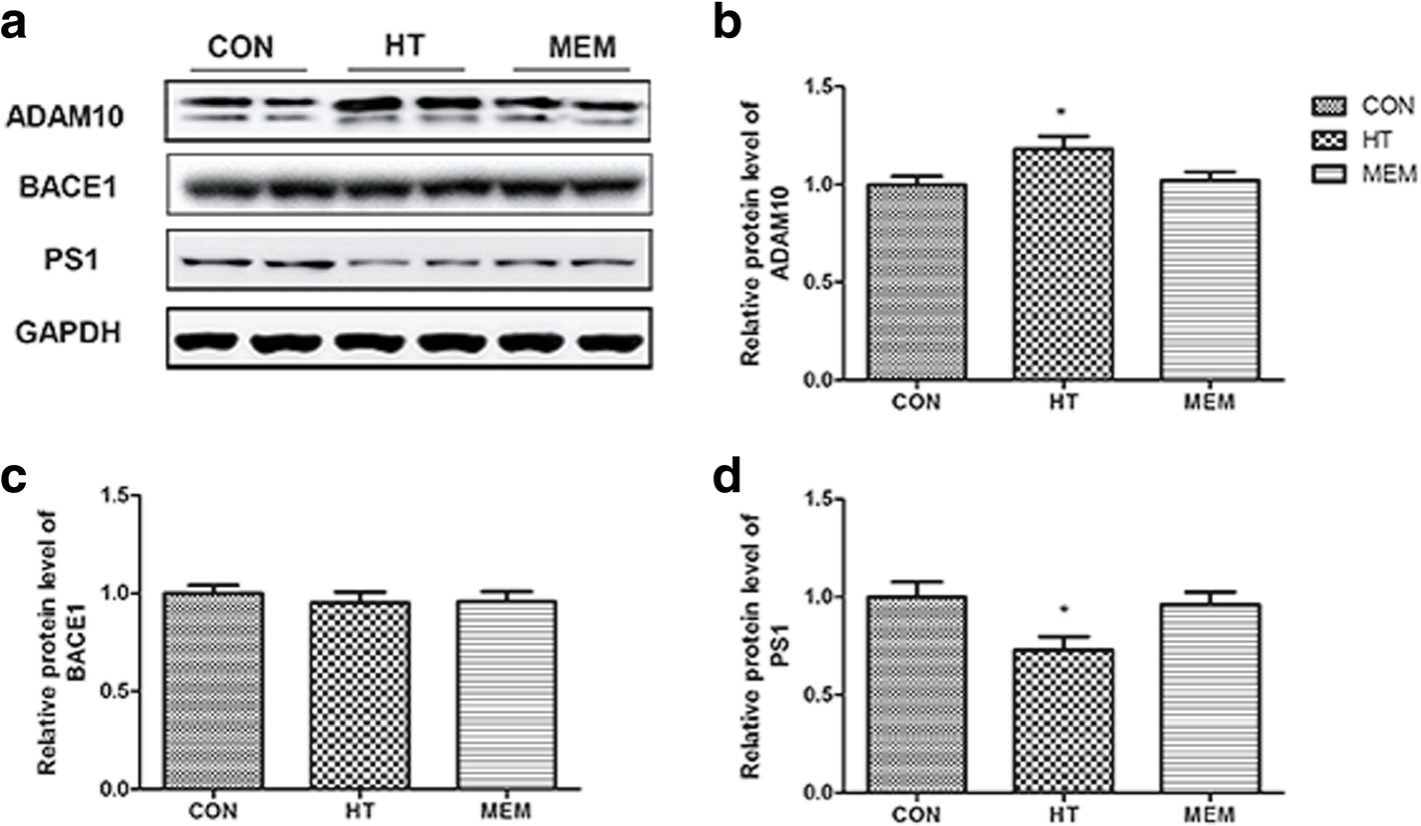
Effects of HT on the enzyme activities of α-secretase, β-secretase, and γ-secretase. **a** The protein levels of ADAM10, Beta-secretase 1 (BACE1) and PS1 were measured by Western blotting in the different groups. **b** Quantitative analysis of ADAM10 expression. *P < 0.05 versus control group; **c** Quantitative analysis of BACE1 expression. **d** Quantitative analysis of PS1 expression. *P < 0.05 versus control group. All data are expressed as the means ± SEM, Significant differences *P < 0.05 compared to the control group, *n* = 6 per group

We determined whether HT has the potential to modify the enzymatic activities of α-secretase, β-secretase, and γ-secretase. We confirmed the effects of HT on Aβ production in APP/PS1 mice (Fig. 8). To further determine the effects of HT on these enzymes, we checked the catalytic activities of these enzymes in brain samples from APP/PS1 mice treated with HT (0.25 g/kg) and MEM (5 mg/kg) for 4 weeks. In this study, the results showed that HT can affect α-secretase (ADAM10) and γ-secretase (PS1) (P < 0.05) (Fig. 8a, b and d). In addition, HT did not affect β-secretase (BACE1) (*P* > 0.05) (Fig. 8c). These data suggest that HT treatment reduces Aβ production, affecting the activities of APP processing enzymes, including ADAM10 and PS1. However, there was no difference between saline-treated and MEM-treated mice in APP processing enzyme activity (Fig. 8).

## Discussion

AD is the most common neurodegenerative disorder in aging populations worldwide. Aβ-induced synaptic dysfunction and loss, which is associated with memory deficits, reduction of synapses, and decreases in synaptic markers occurs in the early and late stages of AD [31]. Aβ is very important in the pathogenesis of AD. Aβ deposition form senile plaques damages synaptic function and eventually leads to neuronal death and cognitive decline. Inhibition of Aβ production is considered to be a potential strategy in AD treatment [32, 33]. There are three main mechanisms of reducing Aβ deposition, i.e., reducing Aβ production [34]; increasing Aβ degradation [35]; and immune clearance of Aβ [36].

APP/PS1 transgenic mice are a typical AD animal model. This model has abnormal behavior and Aβ production [37]. The 6-7 month APP/PS1 mice have memory deficits, learning disabilities and Aβ deposition [38, 39]. Therefore, we choose 6–7-month-old APP/PS1 mice as our experimental animals. The MWM test is a classic behavioral experiment that can test spatial memory. We used escape latency to observe spatial memory and crossing platform frequency and swimming speed to observe working memory. We found that HT can improve spatial memory and working memory in APP/PS1 transgenic mice.

LTP was first observed by Terje Lømo in 1966 in the Oslo, Norway, laboratory of Per Andersen [40]. In neuroscience, LTP constantly enhances synapses based on the recent pattern of activity. LTP is a well-characterized form of synaptic plasticity that fulfils many of the criteria for a neural correlate of memory [41]. In a 2003 study, Rowan et al. proposed one model to explain the impact of AD on LTP [42]. Hippocampal LTP impairment may lead to cognitive decline in early AD. In turn, AD may also damage LTP by a different Aβ mechanism. In our experiments, we found that HT can reverse LTP impairment in the hippocampus of APP/PS1 mice.

Our experiments with APP/PS1 mice showed that pretreatment with HT can reduce Aβ deposition in the brain of APP/PS1 mice. According to the Aβ cascade hypothesis, Aβ is a direct initiation factor that causes a series of pathological processes in AD. The amyloid precursor protein (APP) undergoes constitutive shedding by a protease activity called α-secretase. Three enzymes belong to a disintegrin and metalloproteinase (ADAM) family, ADAM9, ADAM10 and ADAM17. This is considered an important mechanism preventing the generation of the Alzheimer’s disease Aβ peptide (Aβ) [43, 44]. ADAM10 is a member of the ADAM family, which is thought to be a constitutive α-secretase during amyloid-beta protein precursor (AβPP) cleavage. Studies have demonstrated the beneficial role of ADAM10 in reducing the pathologic damage of Alzheimer’s disease (AD) [45, 46]. It has been observed that both ADAM10 localization and its activity are reduced in synapses in the brains of AD patients, especially in hippocampus. Therefore, given the synaptotoxic nature of Aβ and the role of ADAM10 in reducing Aβ generation, an appropriate level of ADAM10 in synapses is indispensable in regulating Aβ activity and maintaining normal synaptic function [47]. Our study showed that HT can increase the expression level of ADAM10. γ-secretase is an aspartyl protease that cleaves a few number of substrates within the membrane environment [48]. It contains at least four components that are required for enzymatic activity: the catalytic component presenilin (PS1 or PS2), anterior pharynx-defective-1 (Aph-1), presenilin enhancer-2 (Pen-2) and the essential cofactor nicastrin [49, 50]. Inactivation of PS1 in APP transgenic mice results in a high accumulation of APP-CTFs at the front of the synaptic front, which could lead to impaired synaptic plasticity and long-term memory deficits [51]. In addition, memory deficiencies in APP/PS1 transgenic mice can be treated by genetic inactivation of PS1, which is one method to effectively reduce Aβ accumulation and prevent plaque deposition [52]. Therefore, a strategy for AD treatment may include the selective increase of α-secretase or γ secretase activity reduction to reduce Aβ production. The results show that HT can reduce the deposition of Aβ, reduce the formation of APP, and promote the degradation of IDE. HT can regulate the α and γ degradation pathway of APP at the same time. ADAM10 is thought to be a constitutive α-secretase during amyloid-beta protein precursor (AβPP) cleavage. HT can improve dementia through regulating ADAM10 expression. However, the underling molecular mechanisms are still not well established. Study showed that ADAM10 expression can be mediated by AMPK/SIRT1 manner [20]. Additional, Sirtuin type 1 (SIRT1) upregulated ADAM10 gene expression via coactivation of the retinoic acid receptor (RAR) transcription factor, leading to reduced production of Aβ and plaques in mice model [53]. In further research, we plan to explore the relationship between HT and SIRT1, in order to illustrate the possible mechanism of HT in treatment of AD.

TCM is the traditional medicine of the Chinese nation that maintains the nation’s reproduction and health; it provides hope for AD treatment. TCM composition is complex, which leads to several significant effects [54] and may possess multi-target treatment for disease. HT consists of several medicinal herbs, including *Ligusticum chuanxiong hort (Umbelliferae), Evodia rutaecarpa (Juss.) Benth, Carthamus tinctorius L.* etc. Clinical reports showed that HT could improve the cognitive function [14, 15]. Results confirmed that there are mainly three compounds included in the HT, as ferulic acid, onjixanthone I and albiflorin [7], especially FA has an effect on the treatment of Alzheimer’s disease [55]. Since from Chinese medicine theory, HT contains many herbs which have function of promoting brain smart, resuscitation and these effects may be could improve cognitive function in AD patients. In this study, we found that HT can improve the memory disorders in APP/PS1 mice via enhancing partially the synaptic plasticity and reducing Aβ deposition.

## Conclusion

This study demonstrates that HT has multiple protective effects in APP/PS1 mice, such as the recovery of synaptic ultrastructure, improved memory defects, and reduced Aβ deposition through promoting its degradation and decreasing its generation. Thus, our study demonstrates that HT may be a novel approach to the prevention and treatment of memory disorders.

## Additional files

Additional file 1: **Figure S1.** Memory testing in APP/PS1 transgenic mice and wild-type mice. (A) APP/PS1 mice showed increased escape latency in hidden platform test of water maze compared to WT mice. (B,C) APP/PS1 mice showed less time spent in the target quadrant and less crossing platform time in probe test of water maze (D) APP/PS1 mice showed longer latency to target than WT mice. (TIF 913 kb)

Additional file 2: **Figure S2.** Memory testing in APP/PS1 transgenic mice with different dose of HT. (A) The number of platform crossings time beween different dose of HT during the probe trial. (B) The latency to target between different dose of HT in APP/PS1 mice APP/PS1 mice. (C) The time in the target quadrant. (D) The latency to escape to a submerged platform. (TIF 2232 kb)

## Abbreviations

AD: Alzheimer’s disease
ADAM: A disintegrin and metalloproteinase
Aph-1: Pharynx-defective-1
Aβ: Amyloid-β (Aβ)
AβPP: Amyloid-beta protein precursor
CON: Control
EPSP: Excitatory postsynaptic potential
HT: Huatuo Zaizao pill
IDE: Insulin-degrading enzyme
LTP: Long-term potentiation
MWM: Morris water maze
NOR: Novel object recognition
OFT: Open field test
Pen-2: Presenilin enhancer-2
