# Supplementary figures and images for: Huatuo Zaizao pill ameliorates cognitive impairment of APP/PS1 transgenic mice by improving synaptic plasticity and reducing Aβ deposition
Source: BMC Complement Altern Med. 2018 May 29;18:167. doi: 10.1186/s12906-018-2237-2 (PMC5975403; doi:10.1186/s12906-018-2237-2)

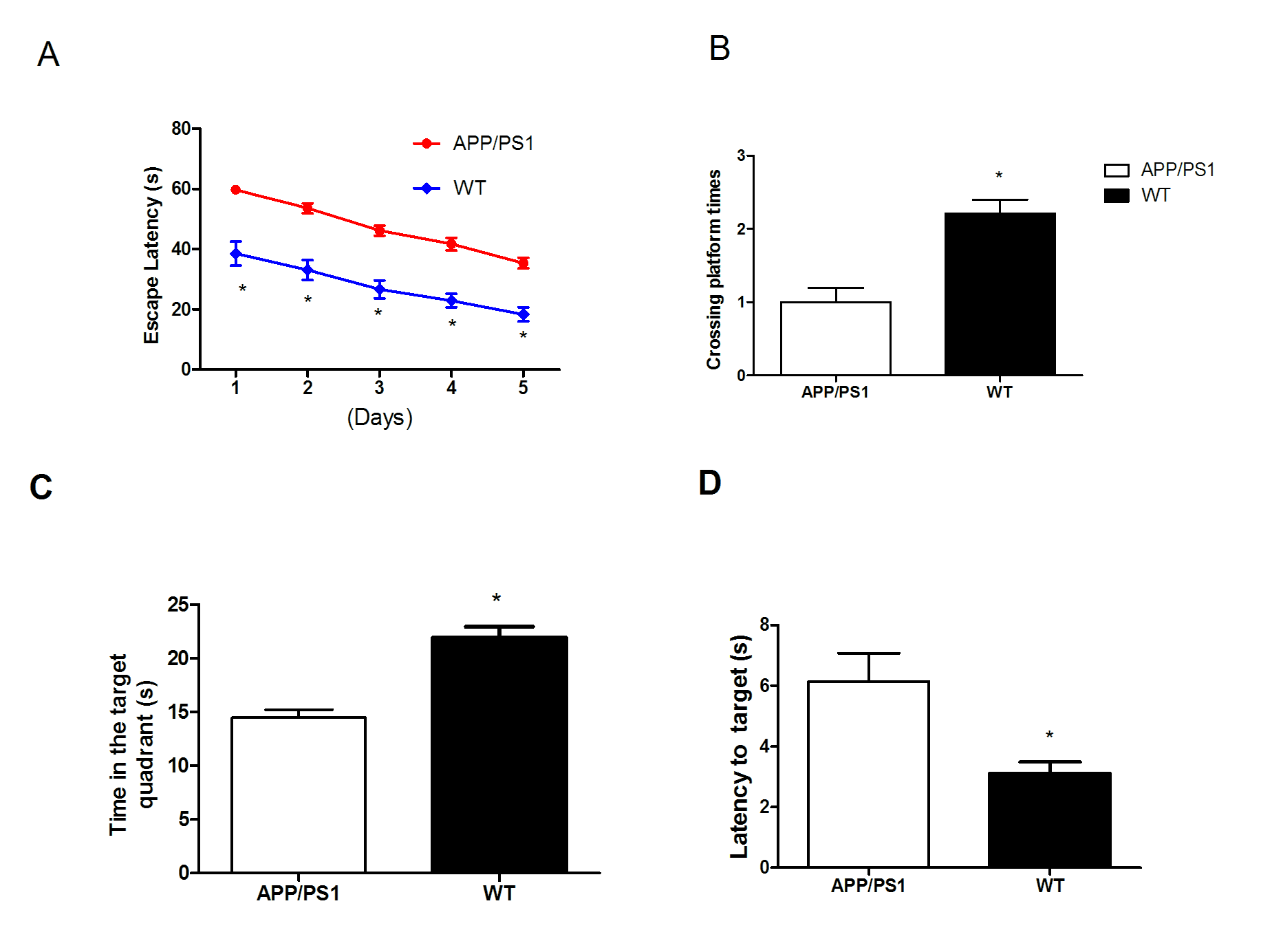

Supplement: Supplementary file 1 — Figure S1. Memory testing in APP/PS1 transgenic mice and wild-type mice. (A) APP/PS1 mice showed increased escape latency in hidden platform test of water maze compared to WT mice. (B,C) APP/PS1 mice showed less time spent in the target quadrant and less crossing platform time in probe test of water maze (D) APP/PS1 mice showed longer latency to target than WT mice. (TIF 913 kb) [file 12906_2018_2237_MOESM1_ESM.tif]

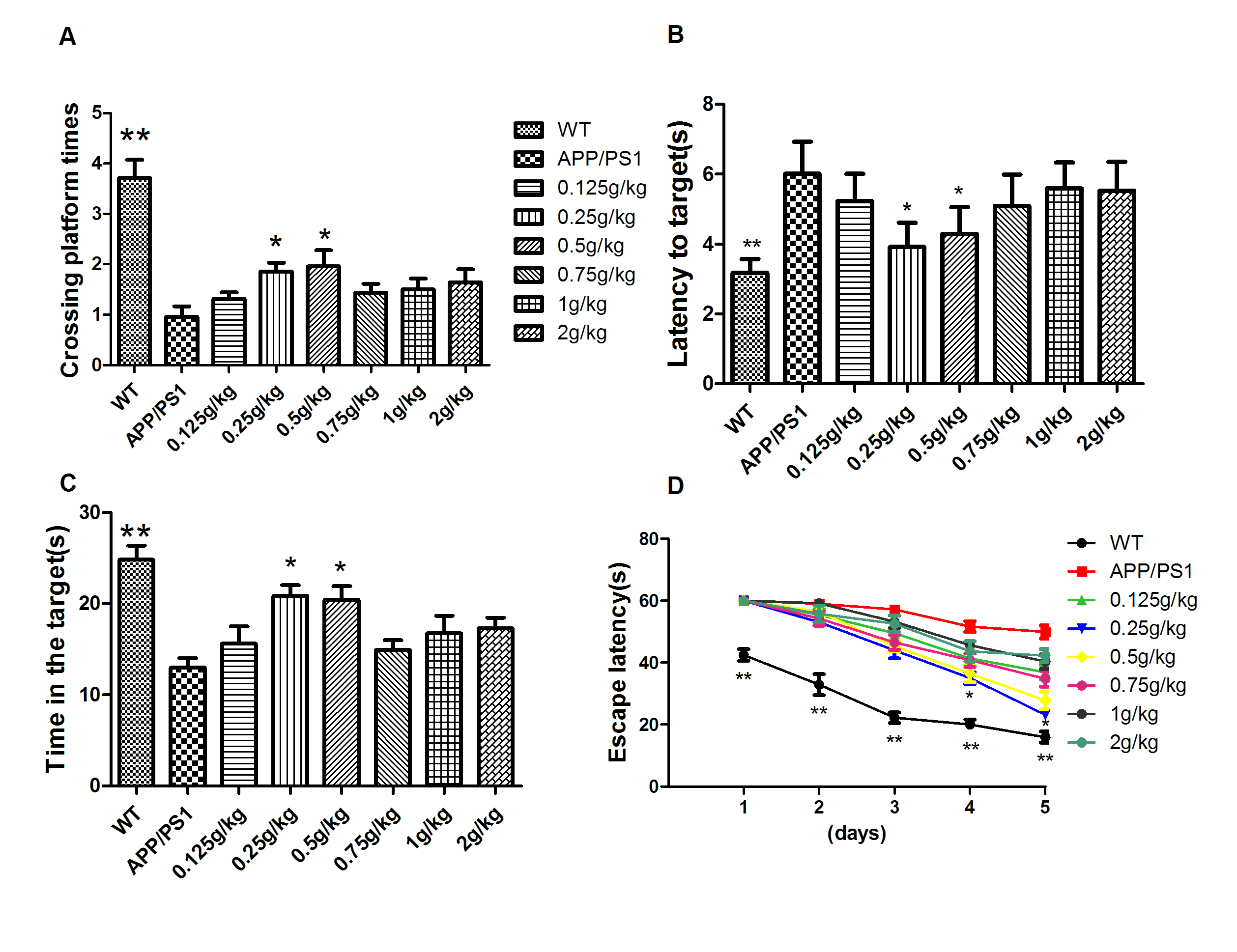

Supplement: Supplementary file 2 — Figure S2. Memory testing in APP/PS1 transgenic mice with different dose of HT. (A) The number of platform crossings time beween different dose of HT during the probe trial. (B) The latency to target between different dose of HT in APP/PS1 mice APP/PS1 mice. (C) The time in the target quadrant. (D) The latency to escape to a submerged platform. (TIF 2232 kb) [file 12906_2018_2237_MOESM2_ESM.tif]
